# Supplementary material for: A CRISPR/Cas9-based enhancement of high-throughput single-cell transcriptomics
Source: Nat Commun. 2025 May 19;16:4664. doi: 10.1038/s41467-025-59880-2 (PMC12089397; doi:10.1038/s41467-025-59880-2)
Supplement: Supplementary file 1 — Supplementary Information [file 41467_2025_59880_MOESM1_ESM.pdf]

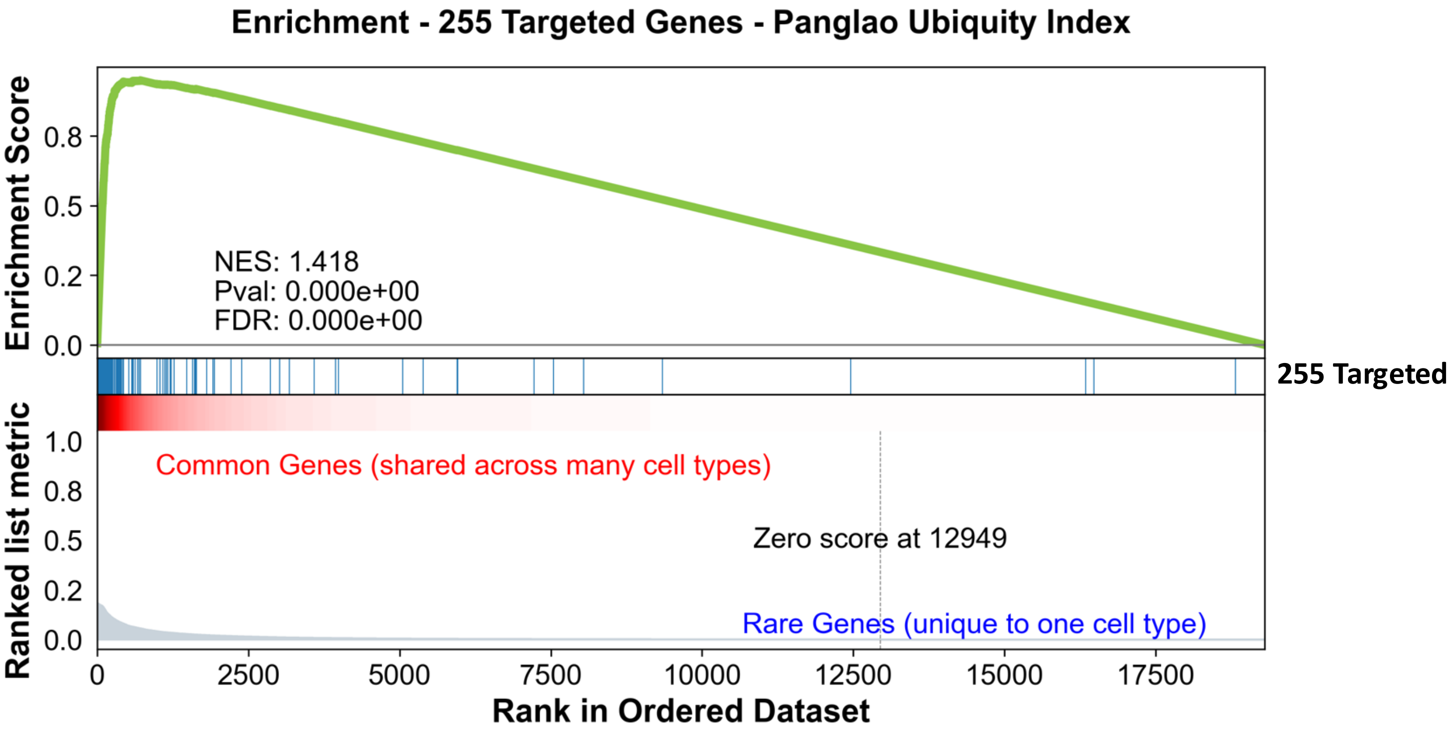

Supplementary Fig. 1: Gene-set enrichment analysis for the 255 gene panel against the PanglaoDB Ubiquitousness Index (UI).

Genes were ranked according to PanglaoDB UI. The location of the 255 targeted genes in the ranked list are indicated as blue dashes. (Top) Enrichment line plot with enrichment score, p- value, and false discovery rate.

<u>**Effect of 255 Targeted scCLEAN Genes on GTEX Bulk Tissues**</u>

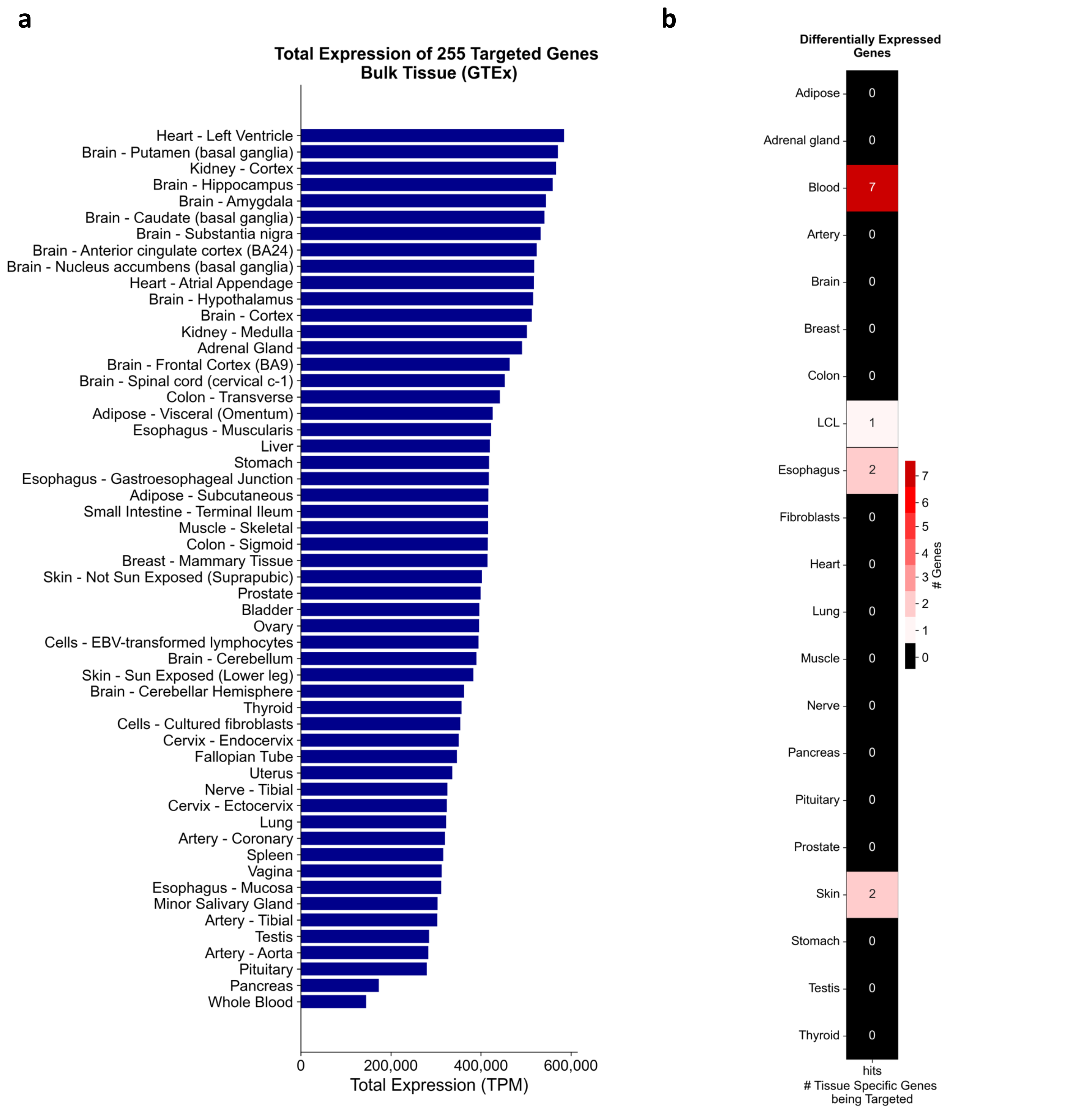

Supplementary Fig. 2: Enrichment of 255 targeted gene panel in GTEx bulk RNAseq tissues.

a. Combined transcripts per million (TPM) of all 255 targeted genes in each GTEx tissue sorted from highest TPM to lowest. **b**. Table summarizing the number of differentially expressed genes within each tissue ($q < 0.99$, Log2FC >= 4) within 255 gene panel.

Total Expression of 255 Targeted Genes across Single Cell Tabula Sapiens

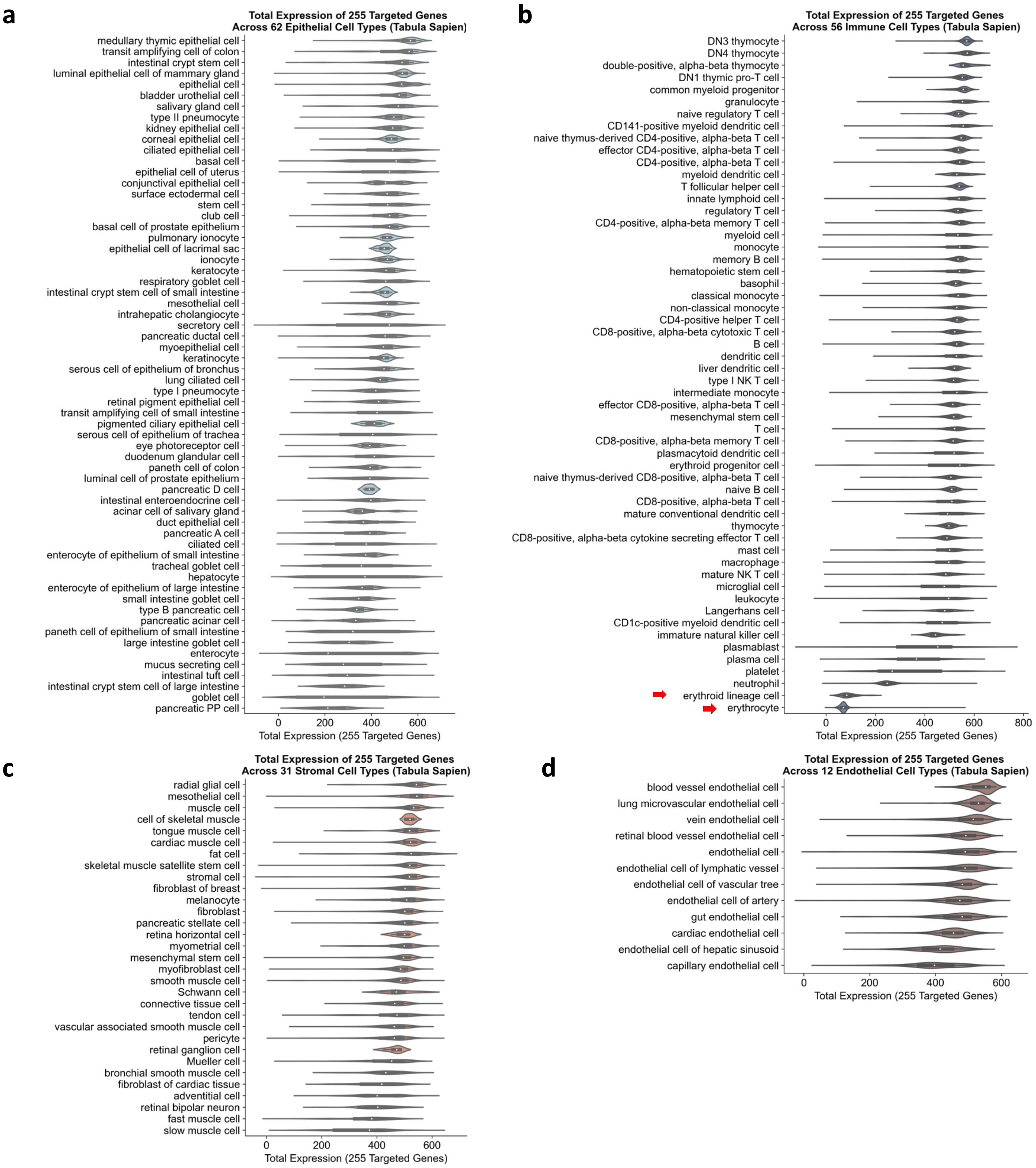

Supplementary Fig. 3: Total expression of the 255 gene panel across the single-cell Tabula Sapiens atlas.

a-d. Cumulative normalized expression of 250 out of the 255 targeted genes (5 were not identified within Tabula Sapiens) across all cell types identified within four categories: 62 epithelial cells (**a**). 56 immune cells (**b**). 31 stromal cells (**c**). 12 endothelial cells (**d**). Red arrows indicate cell types with minimal predicted benefit of scCLEAN.

a

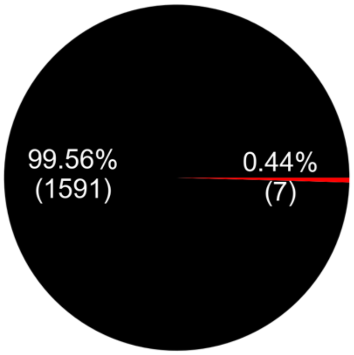

b

Cell Types with Gene Markers Removed (CellMarker 2.0)

Cell Type	# Cell Markers Being Targeted	Total Cell Markers	% Total being Targeted	Cell Specific Genes being Removed
Exhausted CD8+ T cell	1	17	5.88	PTMS
Oligodendrocyte	1	29	3.45	NME2
Regulatory T (Treg) cell	1	34	2.94	GABARAP
Mitotic fetal germ cell	1	64	1.56	SNHG5
Ionocyte cell	1	68	1.47	RPS6KC1
Natural killer T (NKT) cell	24	1674	1.43	RPL36,SUMO2,RPS6KB2,SLC25A6,NDUFB2,PSMA7,RPL19,RPS6KB1,COX6C,EDF1,RPL36AL,SLC25A3,ELOB,MZT2B,SRP14,RPS6KA5,ATP5PF,HNRNPA2B1,ATP5F1D,GUK1,RPS19BP1,UBB,CFL1,EIF1
Monocyte	1	72	1.39	RAC1

Supplementary Fig. 4: Cell types (n=1,598) from the CellMarker 2.0 database with cell specific markers within the 255 gene panel.

a. Proportion of cells within CellMarker 2.0 database that contain at-least one cell marker that is being removed with scCLEAN. **b**. Table indicating which cell types are being affected, the number of cell markers removed, the percent of total cell markers, and the corresponding genes.

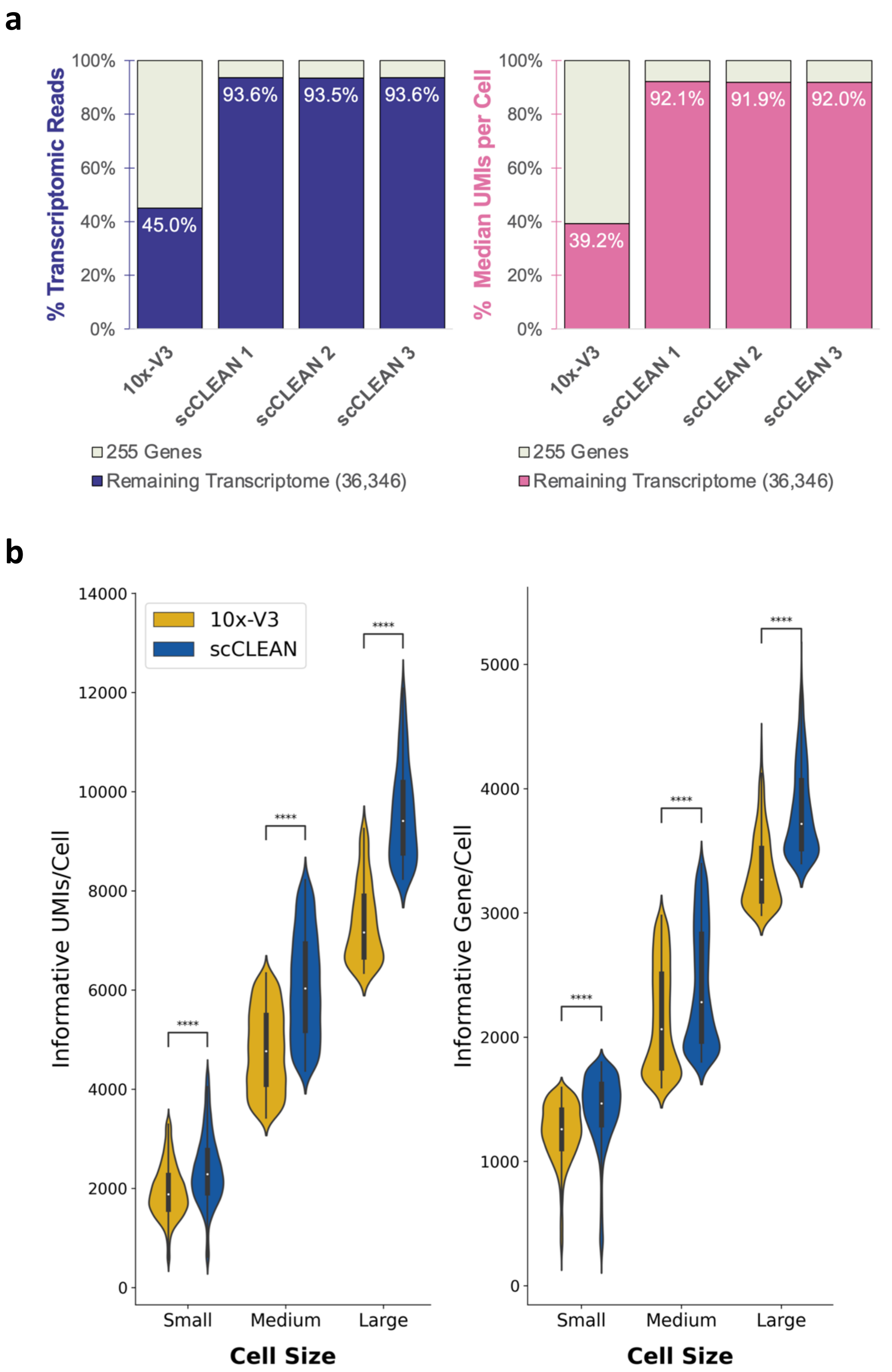

Supplementary Fig. 5: scCLEAN performance metrics in PBMCs.

a. (Top) Percent of aligned transcriptomic reads within the 255 targeted genes or all remaining genes and (Bottom) the percent of median UMIs per cell within the 255 targeted genes or all remaining genes. **b**. (Left) Comparison of the distribution of UMIs per cell associated with the informative genes (excluding the 255 gene panel). Cells grouped into three equivalent sized bins (small, medium, large) representing 1/3 of the range in median UMIs per cell. (Right) Same as in left but comparing the distribution of Genes/cell. Difference quantified using Wilcoxon rank sum with BH correction (q<0.05).

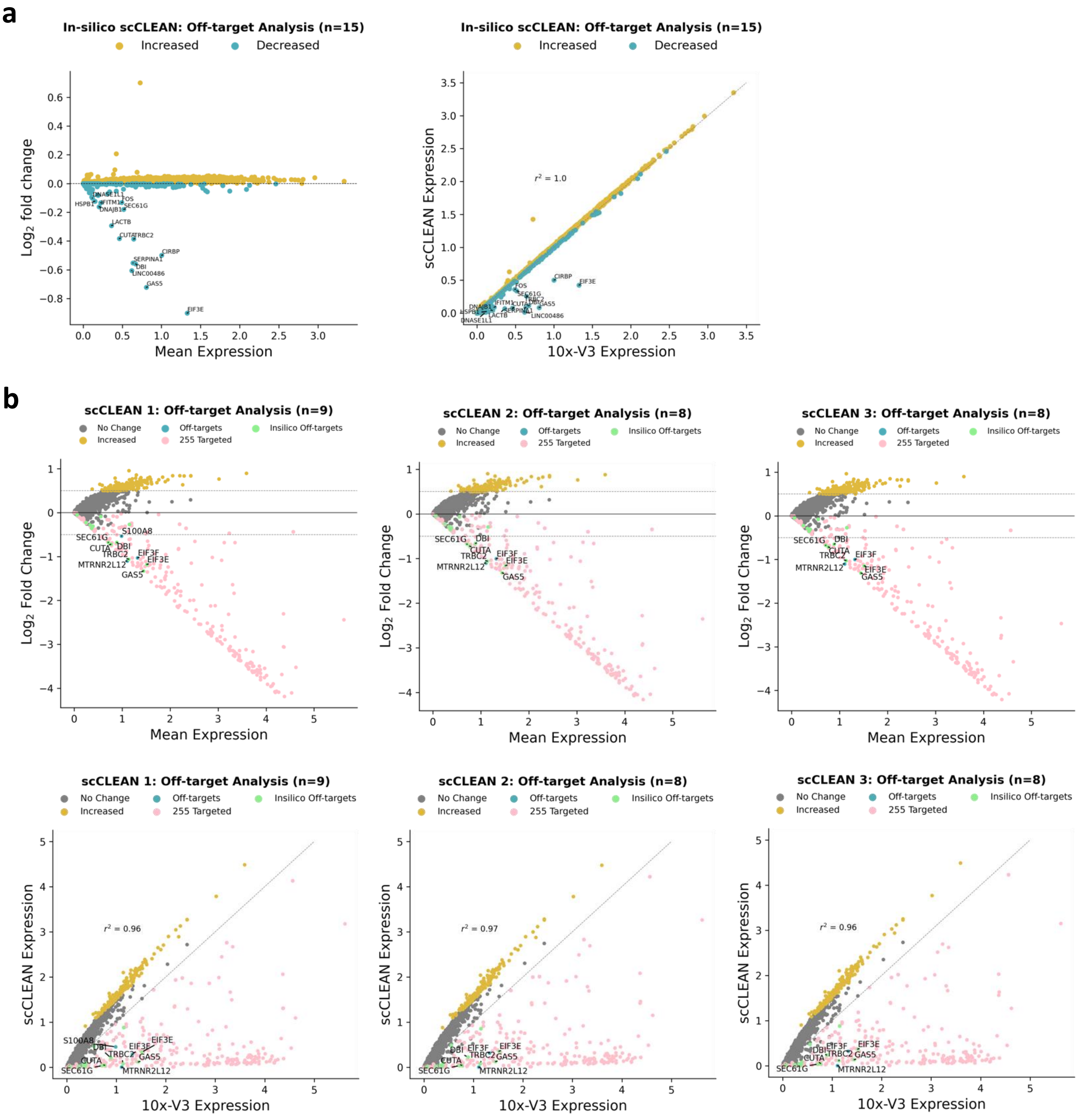

Supplementary Fig. 6: Off-target analysis of scCLEAN utilizing in-silico depletion.

a. (Left) Change in gene expression (Log2 fold change) in the *in-silico* depleted scCLEAN relative to the control 10x-V3 condition from the same cell barcodes. Annotated genes passed a Log2 fold change threshold of -0.1. (Right) Correlation in mean expression (Log2(X+1) normalized). **b**. (Top) Log2 fold change between 3 experimental scCLEAN replicates relative to the control condition: 10x-V3. Annotated genes passed a Log2FC threshold of -0.5. (Bottom) Gene counts correlation between scCLEAN and 10x-V3 (Log2(X+1) normalized). R2 values calculated not including the 255 targeted genes.

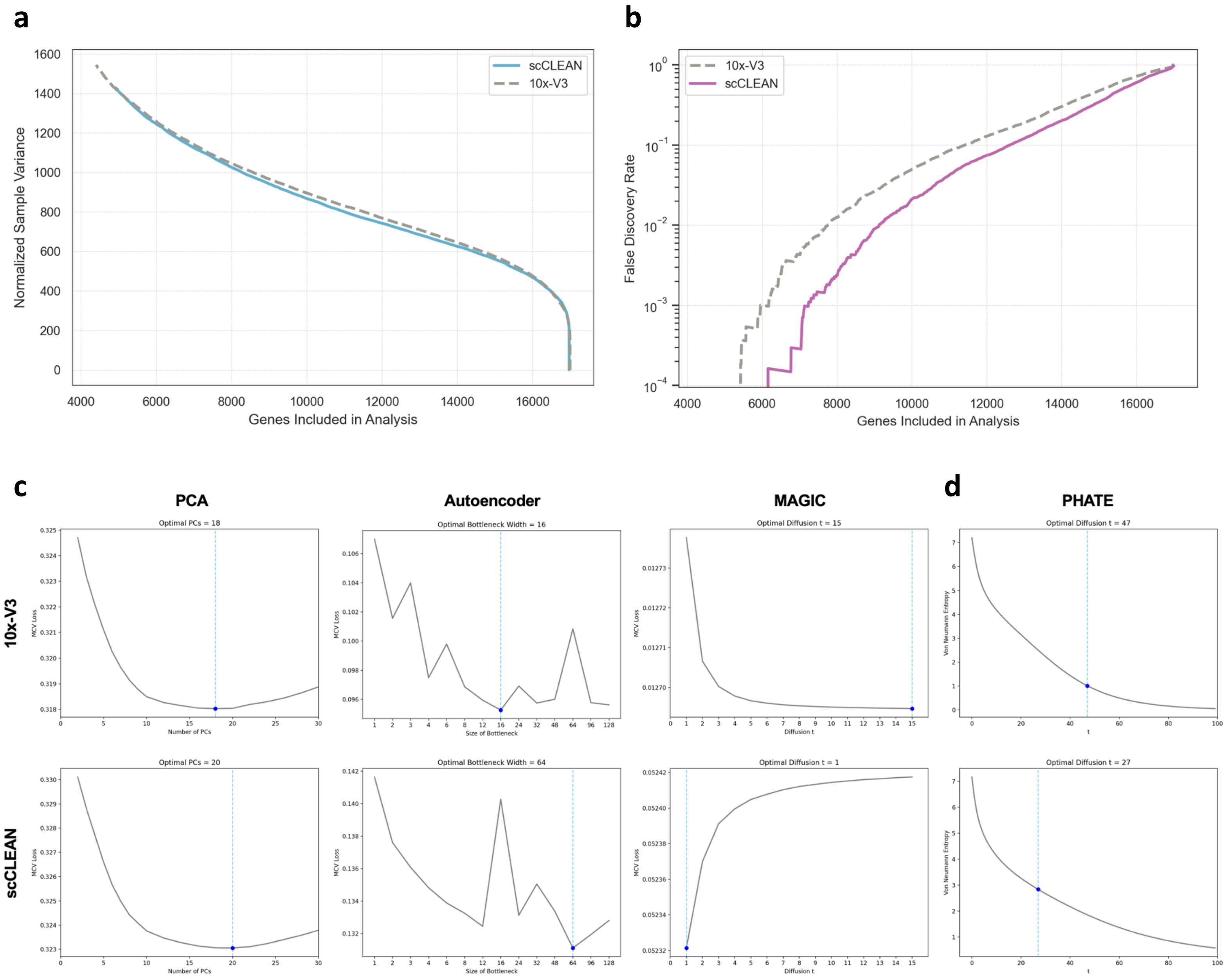

Supplementary Fig. 7: Orthogonal Dimensionality reduction techniques show enhanced signal.

a. Genes included relative to normalized sample variance (10x-V3 = gray, scCLEAN = blue) using Random Matrix Theory (RMT). **b**. RMT to plot the false discovery rate as a function of gene inclusion (10x-V3 = gray, scCLEAN = purple). **c**. Comparing the optimized dimensionality reduction (DR) across various techniques (columns) between 10x-V3 (top row) and scCLEAN (bottom row). The optimal key parameter for each technique was determined using molecular cross validation (MCV loss). (Left) Standard principal component analysis demonstrates increased variance incorporated through DR performance by increasing the number of principal components from 18 (10x-V3) to 20 (scCLEAN). (Middle) Optimized size of the bottleneck layer for a denoising autoencoder for single-cell count data. At the optimal bottleneck width, scCLEAN (width=64) incorporates more signal due to a wider layer than 10x-V3 (width=16). (Right) Proper diffusion parameter, t, for the standard diffusion-based DR algorithm (MAGIC) illustrates a significantly decreased optimized value from 15 (10x-V3) to 1 (scCLEAN). The larger the value, the greater the genetic heterogeneity is smoothed over in an attempt to reduce noise. **d**. DR performance comparison using a diffusion-based algorithm (PHATE) and a separate optimization algorithm based on Von Neuman Entropy. The diffusion component is reduced from 47 (10x-V3) to 27 (scCLEAN).

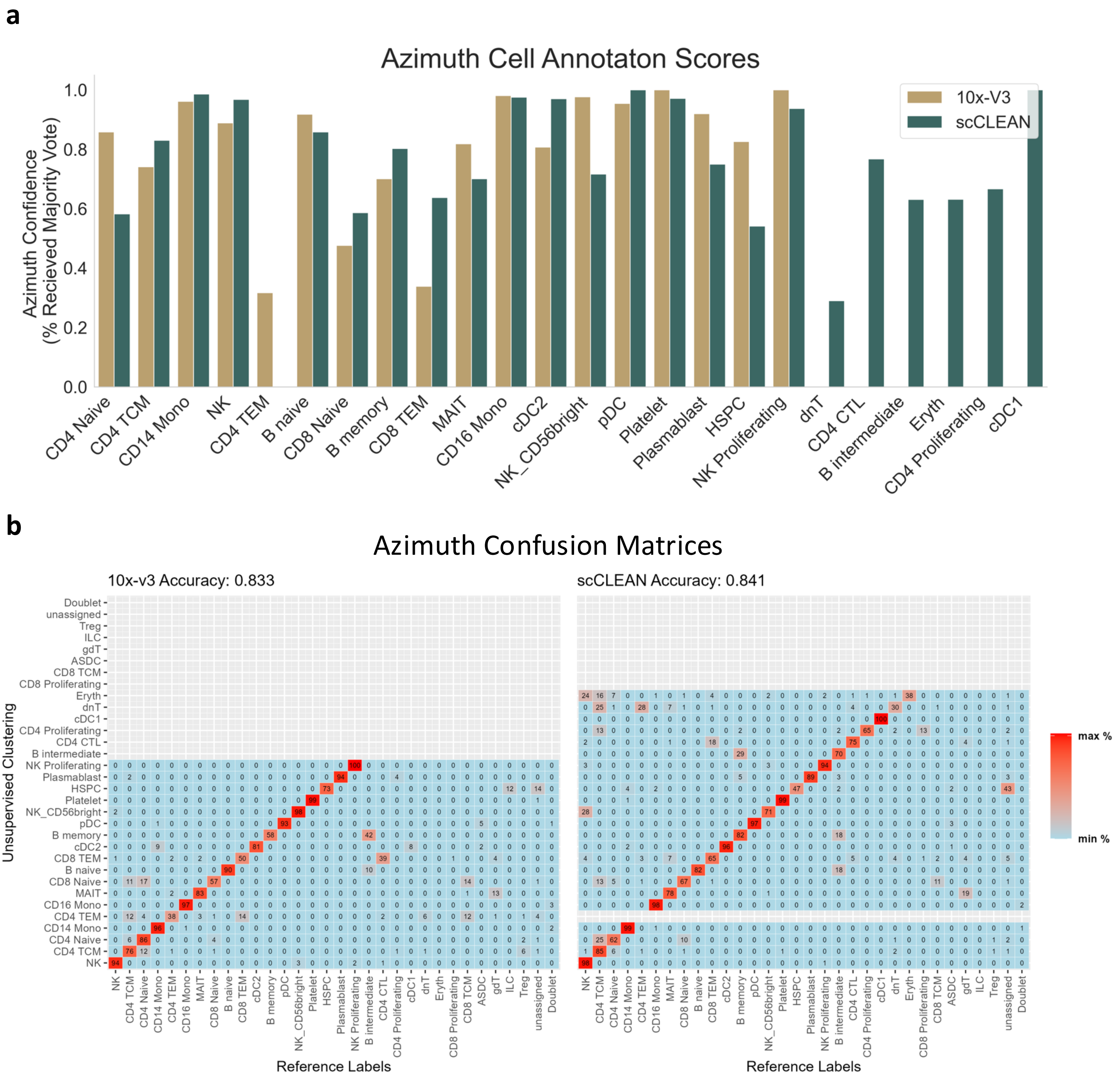

Supplementary Fig. 8: Label transfer metrics for PBMC scRNAseq cell annotation.

a. For all cell types annotated using the Azimuth PBMC reference, confidence in labeling between 10x-V3 (gold) and scCLEAN (green) was measured as the percent of cells within a cell type that were labeled with the majority vote classification. **b**. Correlation matrices comparing 10x-V3 (left) and scCLEAN (right). X-axis represents all potential reference immune cell type labels. Y-axis represents the majority vote label.

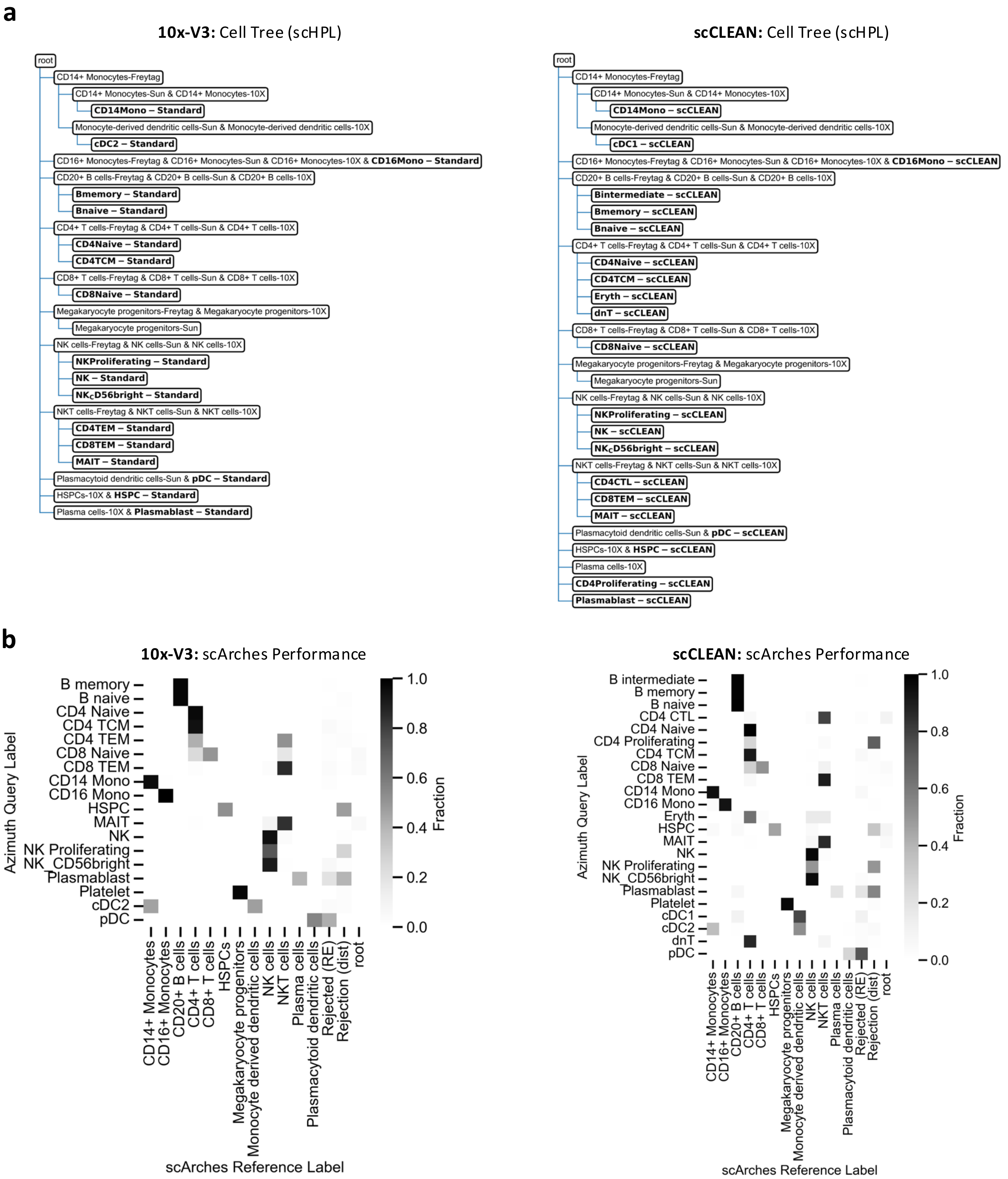

Supplementary Fig. 9: scArches label transfer metrics for PBMC scRNAseq cell annotation.

a. Cell type hierarchy tree (scHPL) between 10x-V3 (left) and scCLEAN (right). Reference dataset consists of PBMC data from three separate publications (Sun, Freytag, 10X). Query data incorporated into the learned tree is bolded. **b.** Heatmap comparison between cell labels and the predicted cell labels from scArches for 10x-V3 (left) and scCLEAN (right).

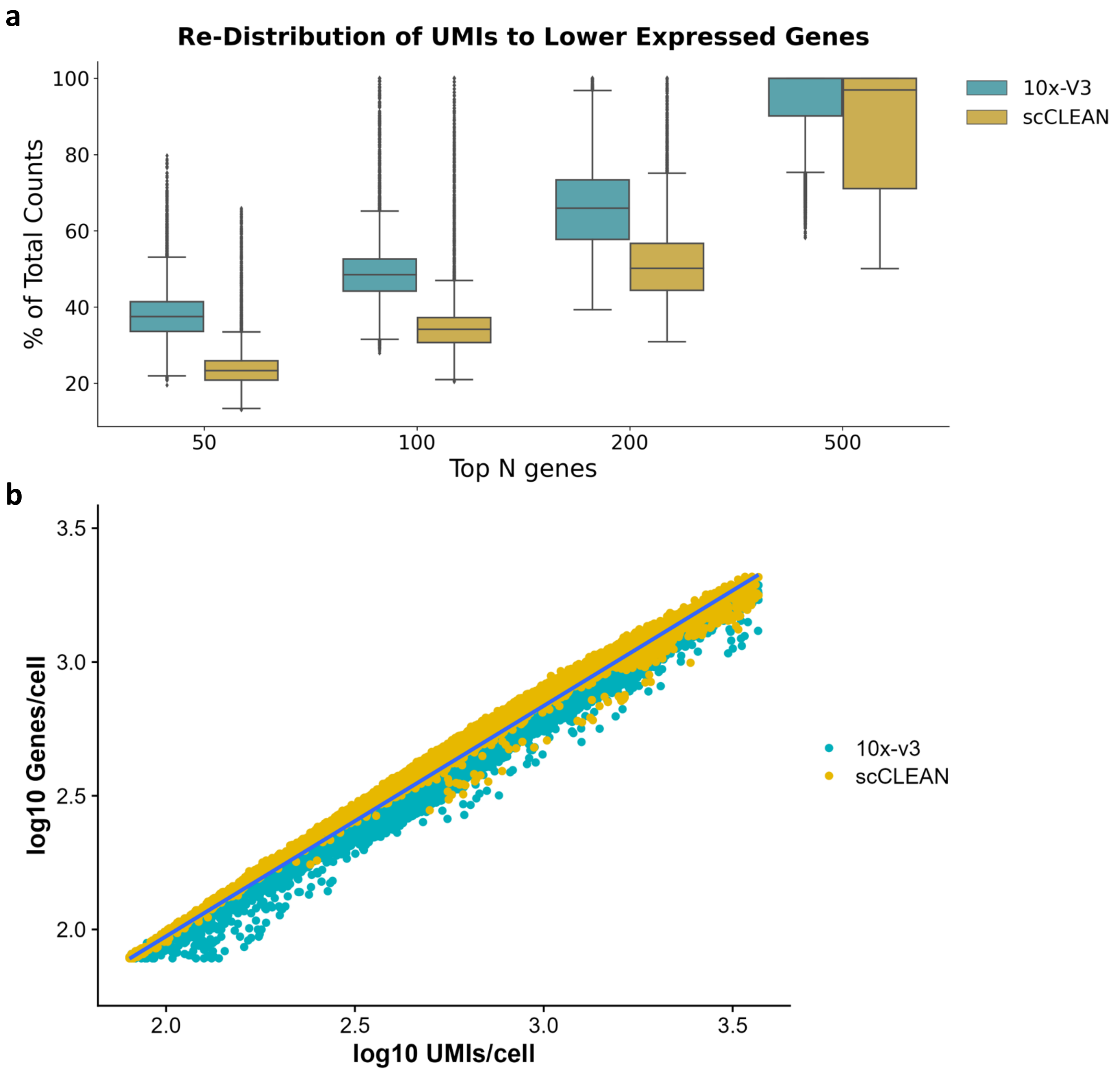

Supplementary Fig. 10: scCLEAN increases library complexity in PBMCs with MAS-seq.

a. Distribution of total UMI counts according to the top 50, 100, 200, 500 genes expressed in 10x-V3 (blue) and scCLEAN (yellow). **b**. Scatter plot of cell complexity. For each cell, the number of genes (log10) plotted as a function of the number of unique UMIs (log10) between 10x-V3 (blue) and scCLEAN (yellow). Blue line indicates linear regression model.

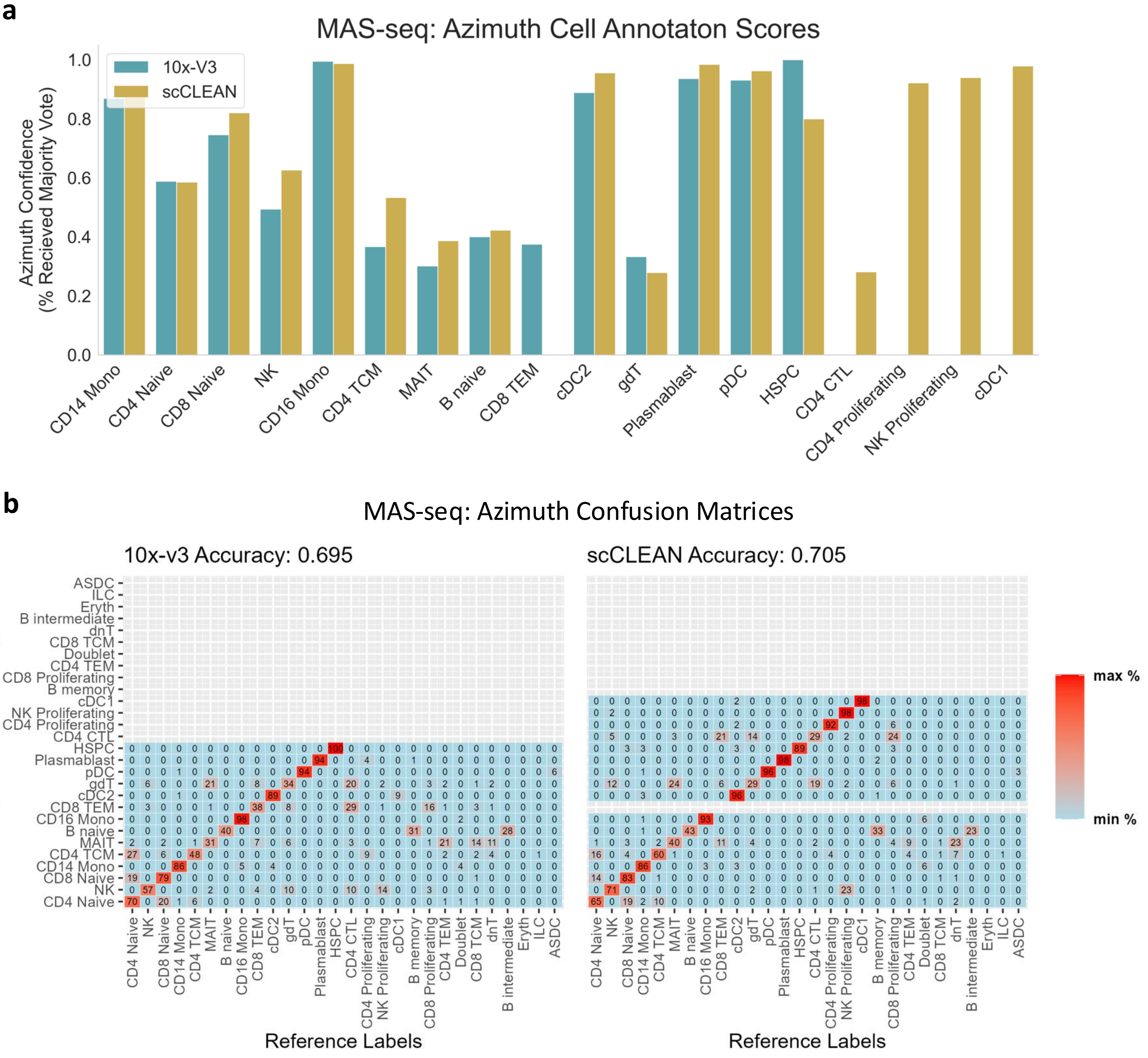

Supplementary Fig. 11: Label transfer performance metrics with MAS-seq.

a. For all cells labeled using the same Azimuth PMBC reference, confidence in labeling between 10x-V3 (blue) and scCLEAN (yellow) was measured as the percent of cells within a cell type that were labeled with the majority vote classification. **b.** Correlation matrices comparing 10x-V3 (left) and scCLEAN (right). X-axis represents all possible immune cell labels in the reference. Y-axis represents the majority vote label.

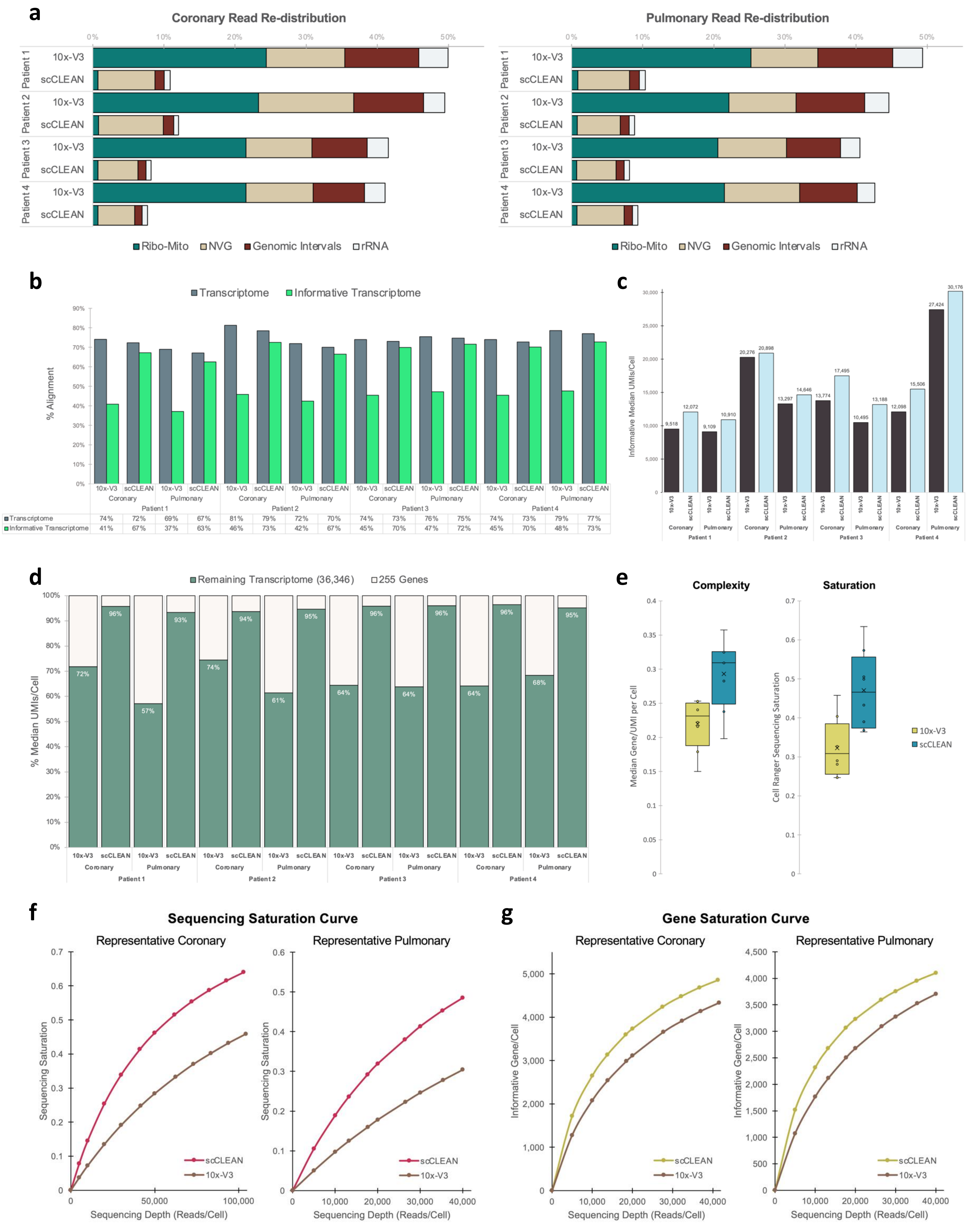

a
Coronary Read Re-distribution
Pulmonary Read Re-distribution
0% 10% 20% 30% 40% 50%
Patient 1
Patient 2
Patient 3
Patient 4
10x-V3
scCLEAN
Ribo-Mito
NVG
Genomic Intervals
rRNA
b
Transcriptome
Informative Transcriptome
% Alignment
Transcriptome 74% 72% 69% 67% 81% 79% 72% 70% 74% 73% 76% 75% 74% 73% 79% 77%
Informative Transcriptome 41% 67% 37% 63% 46% 73% 42% 67% 45% 70% 47% 72% 45% 70% 48% 73%
c
Informative Median UMIs/Cell
9,518 12,072 9,109 10,910 20,276 20,898 13,297 14,646 13,774 17,495 10,495 13,188 12,098 15,506 27,424 30,176
Coronary
Pulmonary
d
Remaining Transcriptome (36,346)
255 Genes
% Median UMIs/Cell
72% 96% 57% 93% 74% 94% 61% 95% 64% 96% 64% 96% 64% 96% 68% 95%
e
Complexity
Saturation
Median Gene/UMI per Cell
Cell Ranger Sequencing Saturation
10x-V3
scCLEAN
f
Sequencing Saturation Curve
Representative Coronary
Representative Pulmonary
Sequencing Saturation
Sequencing Depth (Reads/Cell)
scCLEAN
10x-V3
g
Gene Saturation Curve
Representative Coronary
Representative Pulmonary
Informative Gene/Cell
Sequencing Depth (Reads/Cell)
scCLEAN
10x-V3

Supplementary Fig. 12: scCLEAN Performance on primary VSMC Samples.

a. Read redistribution of 4 targeted regions for removal (Ribo-Mito, NVG, Genomic Intervals, rRNA) within coronary samples (left) and pulmonary samples (right) across all donors. **b**. For all samples, alignment rates to the full transcriptome (dark grey) or the informative transcriptome (lime green) which excludes the 255 gene panel. **c**. Median informative UMIs detected per cell (excluding 255 gene panel) across all donors comparing 10x-V3 (black) to scCLEAN (light blue). **d**. Ratio of all UMIs per cell corresponding with the 255 targeted genes (tan) and the remaining transcriptome (36,346 genes) (green). **e**. Box and whisker plot illustrating the boost in complexity and sequencing saturation with scCLEAN (blue) relative to 10x-V3 (yellow). **f**. Sequencing saturation as a function of sequencing depth comparing scCLEAN (red) and 10x-V3 (brown) illustrating representative coronary (left) and pulmonary (right) samples. **g**. Gene saturation curves depicting informative gene detection (after removal of 255 gene panel) relative to sequencing depth between scCLEAN (yellow) and 10x-V3 (brown) illustrating representative coronary (left) and pulmonary (right) samples.

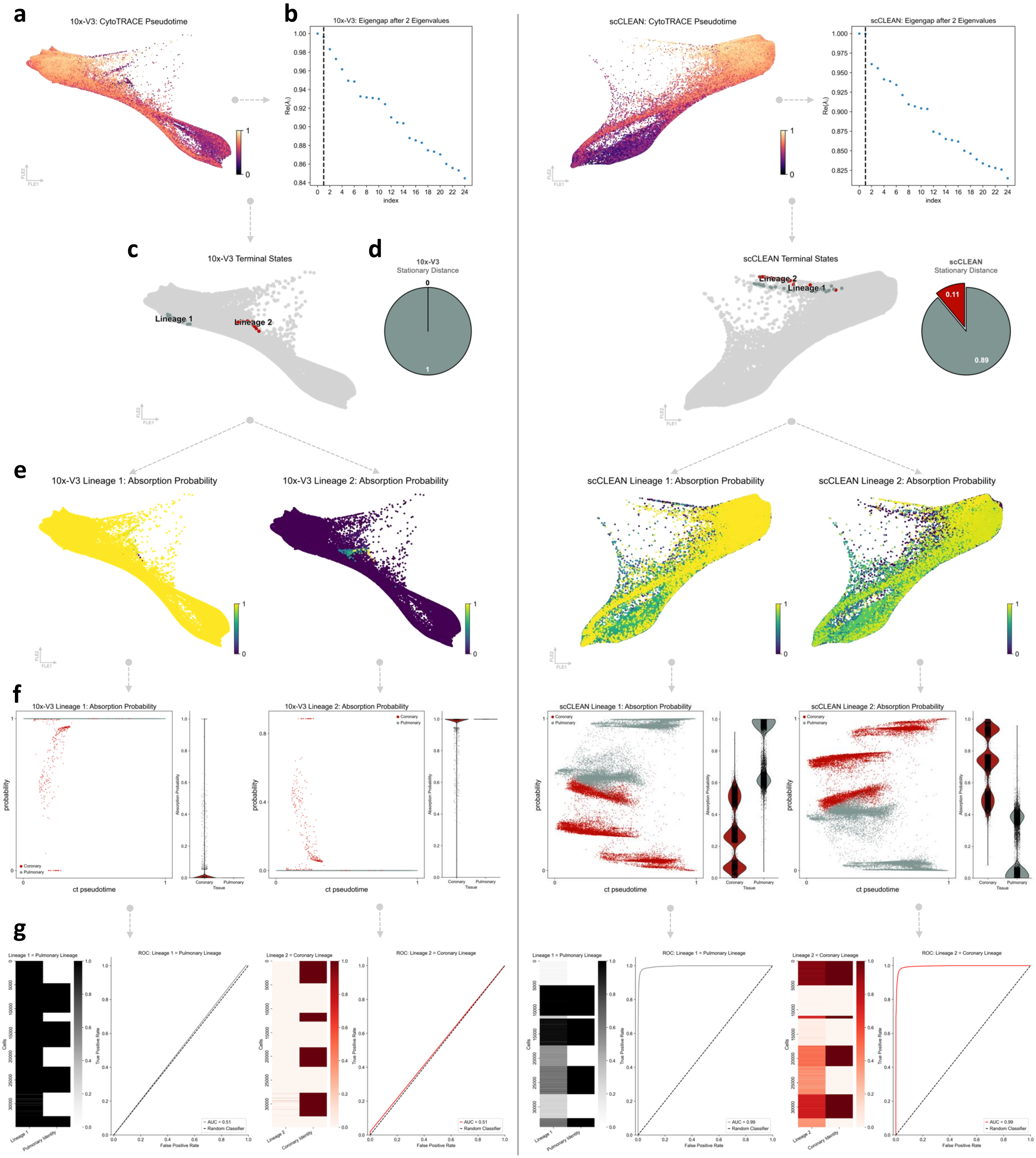
a
10x-V3: CytoTRACE Pseudotime
b
10x-V3: Eigengap after 2 Eigenvalues
Re(λᵢ)
index
scCLEAN: CytoTRACE Pseudotime
scCLEAN: Eigengap after 2 Eigenvalues
c
10x-V3 Terminal States
Lineage 1
Lineage 2
d
10x-V3
Stationary Distance
scCLEAN Terminal States
scCLEAN
Stationary Distance
0.11
0.89
e
10x-V3 Lineage 1: Absorption Probability
10x-V3 Lineage 2: Absorption Probability
scCLEAN Lineage 1: Absorption Probability
scCLEAN Lineage 2: Absorption Probability
f
probability
ct pseudotime
Coronary
Pulmonary
Tissue
Absorption Probability
g
Lineage 1 = Pulmonary Lineage
ROC: Lineage 1 = Pulmonary Lineage
Lineage 2 = Coronary Lineage
ROC: Lineage 2 = Coronary Lineage
Cells
Pulmonary Identity
Coronary Identity
True Positive Rate
False Positive Rate
AUC = 0.51
AUC = 0.99
Random Classifier
FLE1
FLE2

Supplementary Fig. 13: Validation of cardiovascular trajectory analysis.

a-g, Trajectory analysis workflow depicting 10x-V3 (left of line divide) and scCLEAN (right of line divide). **a**. Pseudotime projected on force directed layout plot (FLE), calculated using all genes detected and depicting the transition from early cells (black) to terminal cells (yellow). **b**, Schur decomposition plotting the real components of the top 25 eigenvalues. A gap was calculated after 2 values motivating the calculation of 2 terminal states. **c**. Location of 2 terminal states on FLE projection. **d**. Stationary distance from coarse-grained Markov transition matrix associated with each lineage. **e**. Probability of each cell belonging to each lineage (absorption probability). Yellow illustrates 100% probability of cell-lineage association while dark blue represents 0%. Greater than 99% of cells within 10x-V3 (left) belong to lineage 1, while in scCLEAN (right), 64% of cells correspond to lineage 1 and 36% of cells correspond to lineage 2 (absorption probability > 0.5). **f**. Each cells lineage absorption probability (lineage 1 = left, lineage 2 = right) plotted as a function of each cells position along the differentiation trajectory (ct pseudotime). Coloring reflects whether the cell was derived from a coronary or pulmonary artery. **g**. (Left) heat matrix illustrating the probability of each cell belonging to each lineage (lineage 1 = left, lineage 2 =right) paired side by side with the identity of that cell belonging to each tissue. (Right) Receiver operating characteristic (ROC) depicting the classification performance of identifying each tissue to each lineage. Black indicates pulmonary origin and red indicates coronary origin. Both lineages of 10x- V3 (left of line divide) have an AUC of 0.51 while the corresponding lineages identified with scCLEAN (right of line divide) have an AUC of 0.99.
