# Supplementary material for: A CRISPR/Cas9-based enhancement of high-throughput single-cell transcriptomics
Source: Nat Commun. 2025 May 19;16:4664. doi: 10.1038/s41467-025-59880-2 (PMC12089397; doi:10.1038/s41467-025-59880-2)
Supplement: Supplementary file 2 — Description of Additional Supporting Information [file 41467_2025_59880_MOESM2_ESM.docx]

Supplementary Data Legends.

Supplementary Data 1. Evaluation of abundant and ubiquitous sequences in single cell RNA sequencing across various tissue types. Accession numbers, tissue origin, and publication reference for Sequence Read Archive (SRA) database of the 14 tissues assayed.

Supplementary Data 2. Detailed transcripts used by scCLEAN. Details of the exact transcripts used for removal using scCLEAN including the 10 mitochondrial, 90 ribosomal, and 155 non-variable genes.

Supplementary Data 3. Detailed statistics for box and whisker blots in Figure 1, panel b. Detailed statistics for all groups visualized in Figure 1, panel b.

Supplementary Data 4. Detailed statistics for box and whisker blots in Figure 3, panel d. Detailed statistics for all groups visualized in Figure 3, panel d.
