# Supplementary material for: A CRISPR/Cas9-based enhancement of high-throughput single-cell transcriptomics
Source: Nat Commun. 2025 May 19;16:4664. doi: 10.1038/s41467-025-59880-2 (PMC12089397; doi:10.1038/s41467-025-59880-2)
Supplement: Supplementary file 7 — Reporting Summary [file 41467_2025_59880_MOESM7_ESM.pdf]

nature portfolio

Corresponding author(s): Pandey, Amitabh C

Last updated by author(s): 2024-14-12

Reporting Summary

Nature Portfolio wishes to improve the reproducibility of the work that we publish. This form provides structure for consistency and transparency in reporting. For further information on Nature Portfolio policies, see our Editorial Policies and the Editorial Policy Checklist.

Statistics

For all statistical analyses, confirm that the following items are present in the figure legend, table legend, main text, or Methods section.

n/a	Confirmed	
☐	☒	The exact sample size (*n*) for each experimental group/condition, given as a discrete number and unit of measurement
☐	☒	A statement on whether measurements were taken from distinct samples or whether the same sample was measured repeatedly
☐	☒	The statistical test(s) used AND whether they are one- or two-sided<br>*Only common tests should be described solely by name; describe more complex techniques in the Methods section.*
☐	☒	A description of all covariates tested
☐	☒	A description of any assumptions or corrections, such as tests of normality and adjustment for multiple comparisons
☐	☒	A full description of the statistical parameters including central tendency (e.g. means) or other basic estimates (e.g. regression coefficient) AND variation (e.g. standard deviation) or associated estimates of uncertainty (e.g. confidence intervals)
☐	☒	For null hypothesis testing, the test statistic (e.g. *F*, *t*, *r*) with confidence intervals, effect sizes, degrees of freedom and *P* value noted<br>*Give P values as exact values whenever suitable.*
☐	☒	For Bayesian analysis, information on the choice of priors and Markov chain Monte Carlo settings
☐	☒	For hierarchical and complex designs, identification of the appropriate level for tests and full reporting of outcomes
☐	☒	Estimates of effect sizes (e.g. Cohen's *d*, Pearson's *r*), indicating how they were calculated

Our web collection on statistics for biologists contains articles on many of the points above.

Software and code

Policy information about availability of computer code

Data collection	All analysis software and code used in the study were from publicly available software as described in the manuscript.
Data analysis	Software and tools used in the analysis include:<br>CellRanger v6.2.0<br>bedtools<br>Pegasus (1.5.0)<br>Seurat v.4.1.1<br>scDblFinder<br>ROGUE<br>CellChat<br>CIPR (v.0.1.0)<br>randomly<br>deep embedding for single-cell clustering (DESC)<br>SIMBA (1.1)<br>Circular Consensus Sequencing package v6.4.0<br>samtools v1.12<br>Lima v2.5.0<br>pigeon package v0.1.0.<br>Cell Rank

	Generalized Perron Cluster Cluster Analysis<br>scFATES (0.8.0)<br><br>All parameters and detailed use of packages/software is included in the manuscript and Extended data.

For manuscripts utilizing custom algorithms or software that are central to the research but not yet described in published literature, software must be made available to editors and reviewers. We strongly encourage code deposition in a community repository (e.g. GitHub). See the Nature Portfolio guidelines for submitting code & software for further information.

Data

Policy information about availability of data

All manuscripts must include a data availability statement. This statement should provide the following information, where applicable:

- Accession codes, unique identifiers, or web links for publicly available datasets
- A description of any restrictions on data availability
- For clinical datasets or third party data, please ensure that the statement adheres to our policy

All single-cell sequencing reads, and count matrices are in the process of being submitted to the Gene Expression Omnibus (GSE283554) and additional files at DOI: 10.6084/m9.figshare.26444173. All code is available at https://github.com/acpandey/scCLEAN and available at DOI: 10.5281/zenodo.14079211 Reasonable requests for additional data should be sent to the corresponding author.

Research involving human participants, their data, or biological material

Policy information about studies with human participants or human data. See also policy information about sex, gender (identity/presentation), and sexual orientation and race, ethnicity and racism.

Reporting on sex and gender	All human donor samples (PBMCs and VSMCs) were de-identified to the study investigators and no data on sex or gender was available.
Reporting on race, ethnicity, or other socially relevant groupings	All human donor samples (PBMCs and VSMCs) were de-identified to the study investigators and no data on race, ethnicity, or other social information was available.
Population characteristics	All human donor samples (PBMCs and VSMCs) were de-identified to the study investigators. Data that was provided was that all donors were categorized as young donors and without history of cardiovascular disease for the VSMCs.
Recruitment	All donors were available from Institutional biobank and/or commercially purchases as detailed in the methods.
Ethics oversight	The study was approved by the Institutional Review Board of Scripps Clinic/Scripps Research Institute (IRB-18-7266) and Tulane University (2022-1434-TUHSC).

Note that full information on the approval of the study protocol must also be provided in the manuscript.

Field-specific reporting

Please select the one below that is the best fit for your research. If you are not sure, read the appropriate sections before making your selection.

☒ Life sciences ☐ Behavioural & social sciences ☐ Ecological, evolutionary & environmental sciences

For a reference copy of the document with all sections, see nature.com/documents/nr-reporting-summary-flat.pdf

Life sciences study design

All studies must disclose on these points even when the disclosure is negative.

Sample size	Sample sizes were chosen based on the availability of samples. VSMCs were used for four independent biological donors and matched isolation of coronary and pulmonary VSMCs. PBMCs were from the biobank with pooling of 3 donors.
Data exclusions	No data were excluded for the analysis of this study.
Replication	All data and results were validated with multiple replicates (minimum of three) as well as several orthogonal validation studies to assess consistency of reproducibility of the data.
Randomization	All samples were assayed and there was no scope for randomization in this study.
Blinding	All study investigators were blinded to any identifiable data of the biological samples as per IRB approval. Additionally, initial computational analysis was completed in a blinded fashion without assignment of sample number rather than sample identity.

Reporting for specific materials, systems and methods

We require information from authors about some types of materials, experimental systems and methods used in many studies. Here, indicate whether each material, system or method listed is relevant to your study. If you are not sure if a list item applies to your research, read the appropriate section before selecting a response.

Materials & experimental systems

n/a	Involved in the study
x	Antibodies
	x Eukaryotic cell lines
x	Palaeontology and archaeology
x	Animals and other organisms
x	Clinical data
x	Dual use research of concern
x	Plants

Methods

n/a	Involved in the study
x	ChIP-seq
x	Flow cytometry
x	MRI-based neuroimaging

Eukaryotic cell lines

Policy information about cell lines and Sex and Gender in Research

Cell line source(s)	PBMCs were obtained from the Scripps BioRespository. The VSMCs used in the study were purchased from Cell Applications. All cells were primary culture and below Passage 3.
Authentication	Authentication of the cells were completed by Cell Applications. No authentication was done for PBMCs.
Mycoplasma contamination	Cells were tested and were negative for myocplasama. Testing for the PBMC samples were from Scripps myocplasma core and for the VSMCs was from Cell Applications.
Commonly misidentified lines (See ICLAC register)	No cells from the register were used in the present study.

Plants

Seed stocks	n/a
Novel plant genotypes	n/a
Authentication	n/a
